# Supplementary material for: The human fungal pathogen Aspergillus fumigatus can produce the highest known number of meiotic crossovers
Source: PLoS Biol. 2023 Sep 14;21(9):e3002278. doi: 10.1371/journal.pbio.3002278 (PMC10501685; doi:10.1371/journal.pbio.3002278)
Supplement: S1 Fig — (A) Dotplot comparison of sequence similarity using minimap2 of AfIR974 assembly against reference Af293 assembly. (B) Comparison of AfIR964 against Af293. (C) Comparison of assemblies of 2 parental strains AfIR964 and AfIR974. Data underling this figure can be found at https://doi.org/10.5281/zenodo.8167717. (DOCX) [file pbio.3002278.s001.docx]

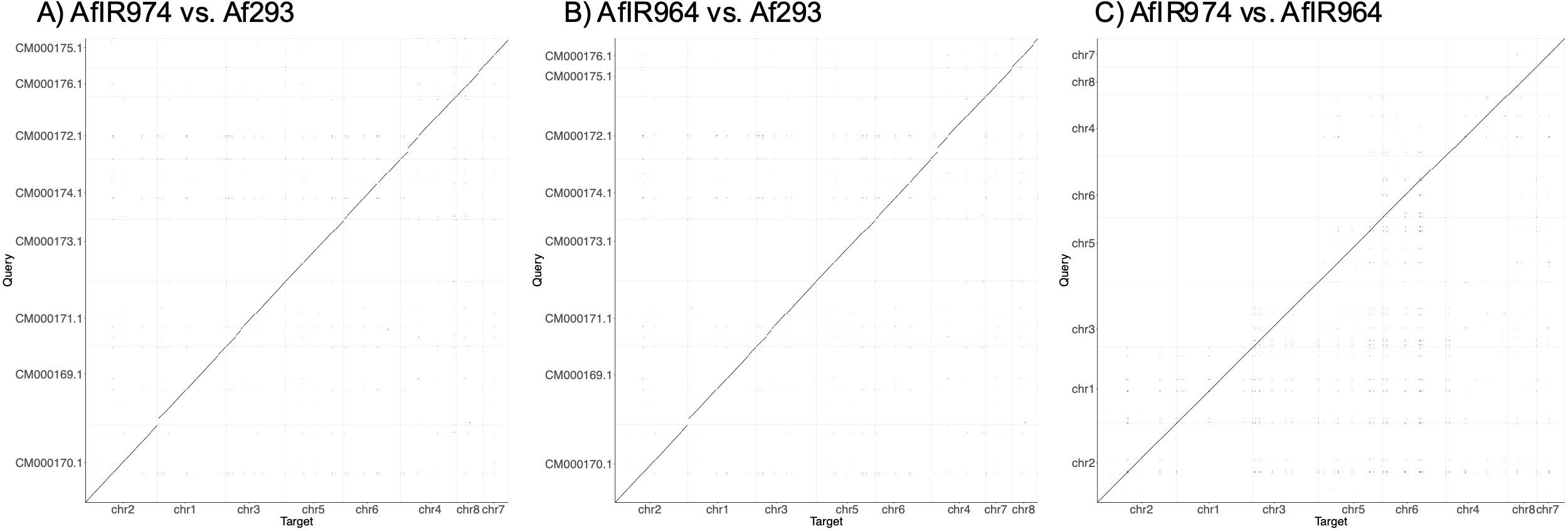
 **Fig. S1: Synteny of two parental strains and reference strain Af293**. **(A)** Dotplot comparison of sequence similarity using minimap2 of AfIR974 assembly against reference Af293 assembly. **(B)** Comparison of AfIR964 against Af293. **(C)** Comparison of assemblies of two parental strains AfIR964 and AfIR974.
