# Supplementary material for: The human fungal pathogen Aspergillus fumigatus can produce the highest known number of meiotic crossovers
Source: PLoS Biol. 2023 Sep 14;21(9):e3002278. doi: 10.1371/journal.pbio.3002278 (PMC10501685; doi:10.1371/journal.pbio.3002278)
Supplement: S2 Fig — (A) Comparison of raw dataset, including presumed gene conversions. Each data point indicates the distance between a pair of adjacent markers, with physical distance shown on a log scale. Density of markers shown by curve for both physical and genetic distances. Note that appreciable recombination between markers is only seen above 100 bp. (B) Comparison of dataset after removal of gene conversions. Data underling this figure can be found at https://doi.org/10.5281/zenodo.8167717. (DOCX) [file pbio.3002278.s002.docx]

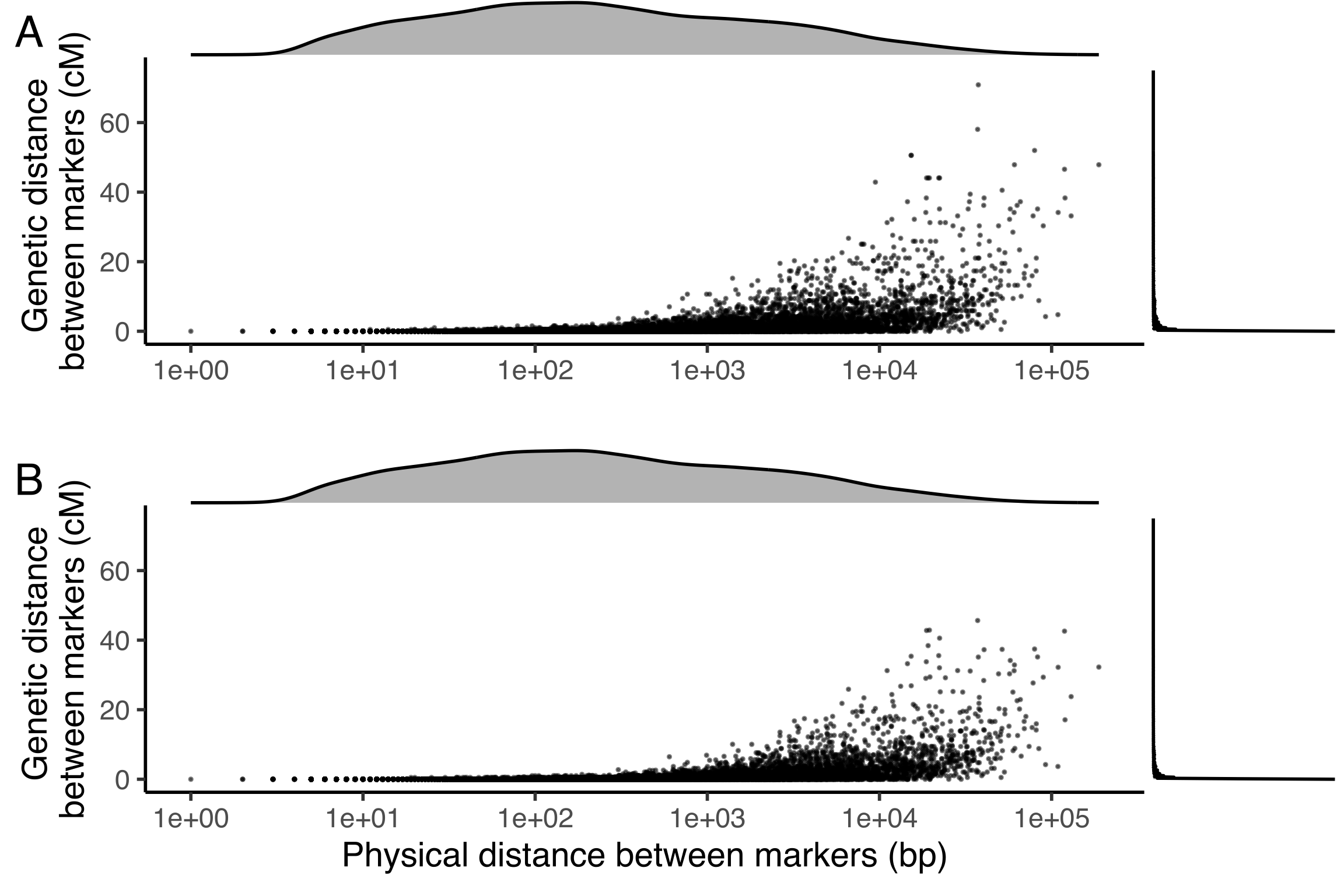


**Fig. S2**: **Comparison of physical and genetic distance between markers. (A)** Comparison of raw dataset, including presumed gene conversions. Each data point indicates the distance between a pair of adjacent markers, with physical distance shown on a log scale. Density of markers shown by curve for both physical and genetic distances. Note that appreciable recombination between markers is only seen above 100 bp. **(B)** Comparison of dataset after removal of gene conversions.
