# Supplementary material for: The human fungal pathogen Aspergillus fumigatus can produce the highest known number of meiotic crossovers
Source: PLoS Biol. 2023 Sep 14;21(9):e3002278. doi: 10.1371/journal.pbio.3002278 (PMC10501685; doi:10.1371/journal.pbio.3002278)
Supplement: S3 Fig — (A–I) Plots of genome-wide observed distances between crossovers (dotted line) and simulated distances between crossovers (solid line). Rows indicate simulations for different genetic map lengths as indicated. Right panel show the same data as the left panel but using a log scale on the x-axis. Data underling this figure can be found at https://doi.org/10.5281/zenodo.8167717. (DOCX) [file pbio.3002278.s003.docx]

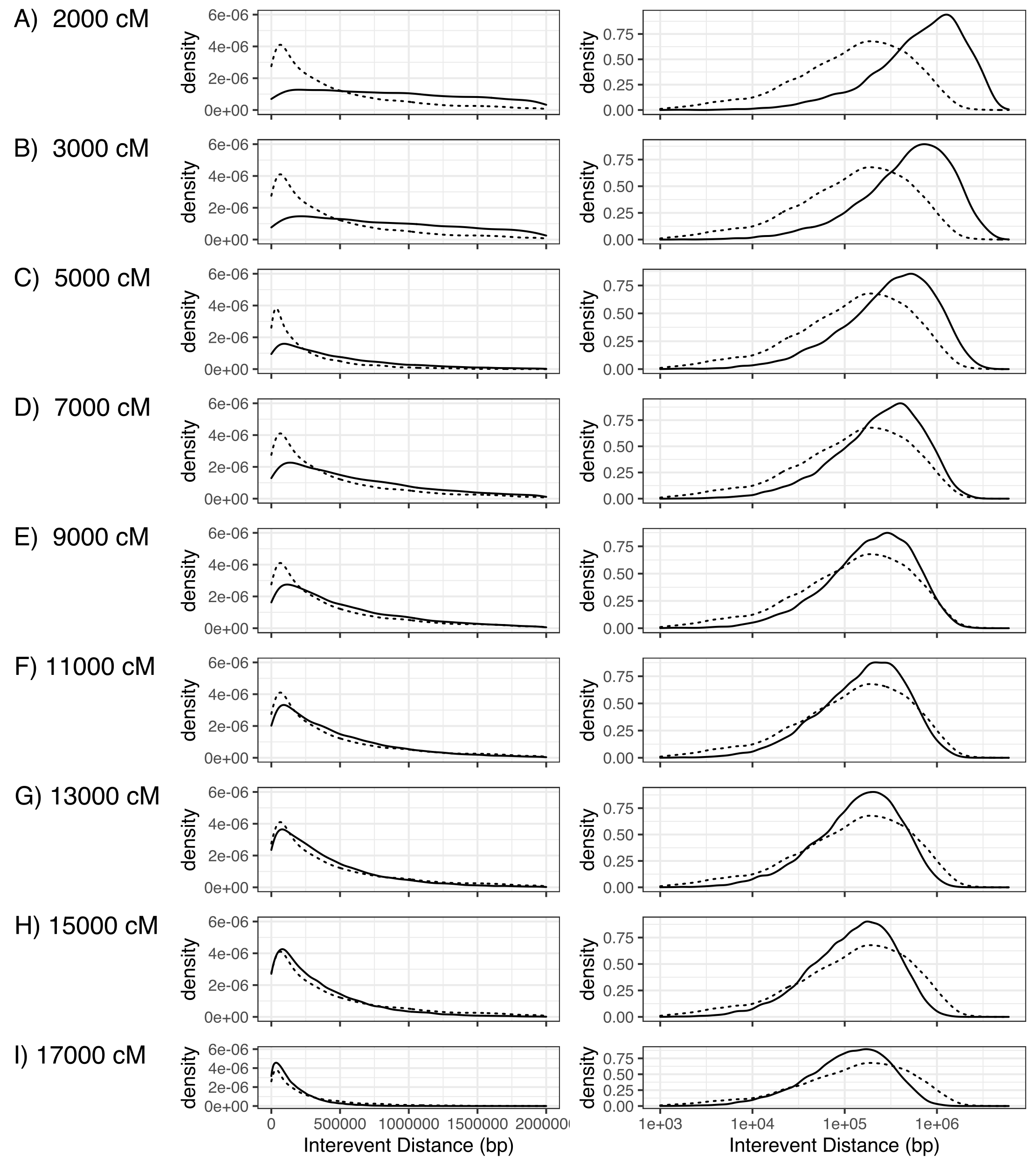


**Fig. S3. Comparison of cleaned dataset against uniformly distributed simulated maps. (A-I)** Plots of genome-wide observed distances between crossovers (dotted line) and simulated distances between crossovers (solid line). Rows indicate simulations for different genetic map lengths as indicated. Right panel show the same data as the left panel but using a log scale on the x-axis.
