# Supplementary material for: The human fungal pathogen Aspergillus fumigatus can produce the highest known number of meiotic crossovers
Source: PLoS Biol. 2023 Sep 14;21(9):e3002278. doi: 10.1371/journal.pbio.3002278 (PMC10501685; doi:10.1371/journal.pbio.3002278)
Supplement: S4 Fig — (A) Small dots indicate values extracted from Stapley and colleagues comparing genome size to genetic map length. Larger triangle indicates A. fumigatus. (B) Similar to A except controlling for chromosome number. Data underling this figure can be found at https://doi.org/10.5281/zenodo.8167717. (DOCX) [file pbio.3002278.s004.docx]

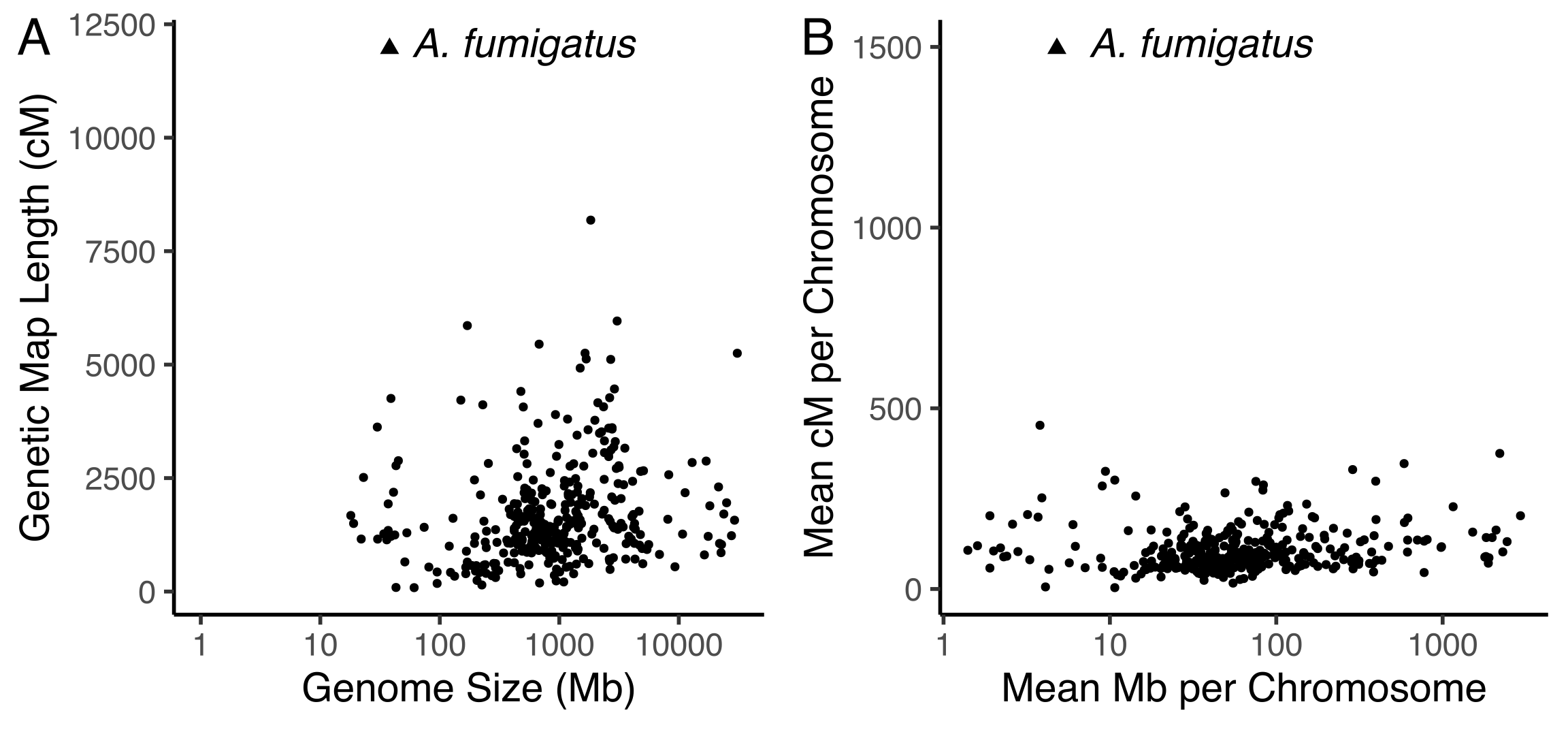


**Fig. S4: Comparison of genetic map of *A. fumigatus* to Stapley et al. 2017 dataset.** **(A)** Small dots indicate values extracted from Stapley et al., 2017, comparing genome size to genetic map length. Larger triangle indicates *A. fumigatus*. **(B)** Similar to A except controlling for chromosome number.
