# Supplementary material for: The human fungal pathogen Aspergillus fumigatus can produce the highest known number of meiotic crossovers
Source: PLoS Biol. 2023 Sep 14;21(9):e3002278. doi: 10.1371/journal.pbio.3002278 (PMC10501685; doi:10.1371/journal.pbio.3002278)
Supplement: S5 Fig — Plots are similar to Fig 2B of the main text but shown for all 8 chromosomes. Dotted line indicates the genome-wide average recombination rate, solid line indicates recombination rate across 50 kb windows. Small vertical hash lines at top of plots indicate marker positions, and large circle indicates estimated centromere position. Data underling this figure can be found at https://doi.org/10.5281/zenodo.8167717. (DOCX) [file pbio.3002278.s005.docx]

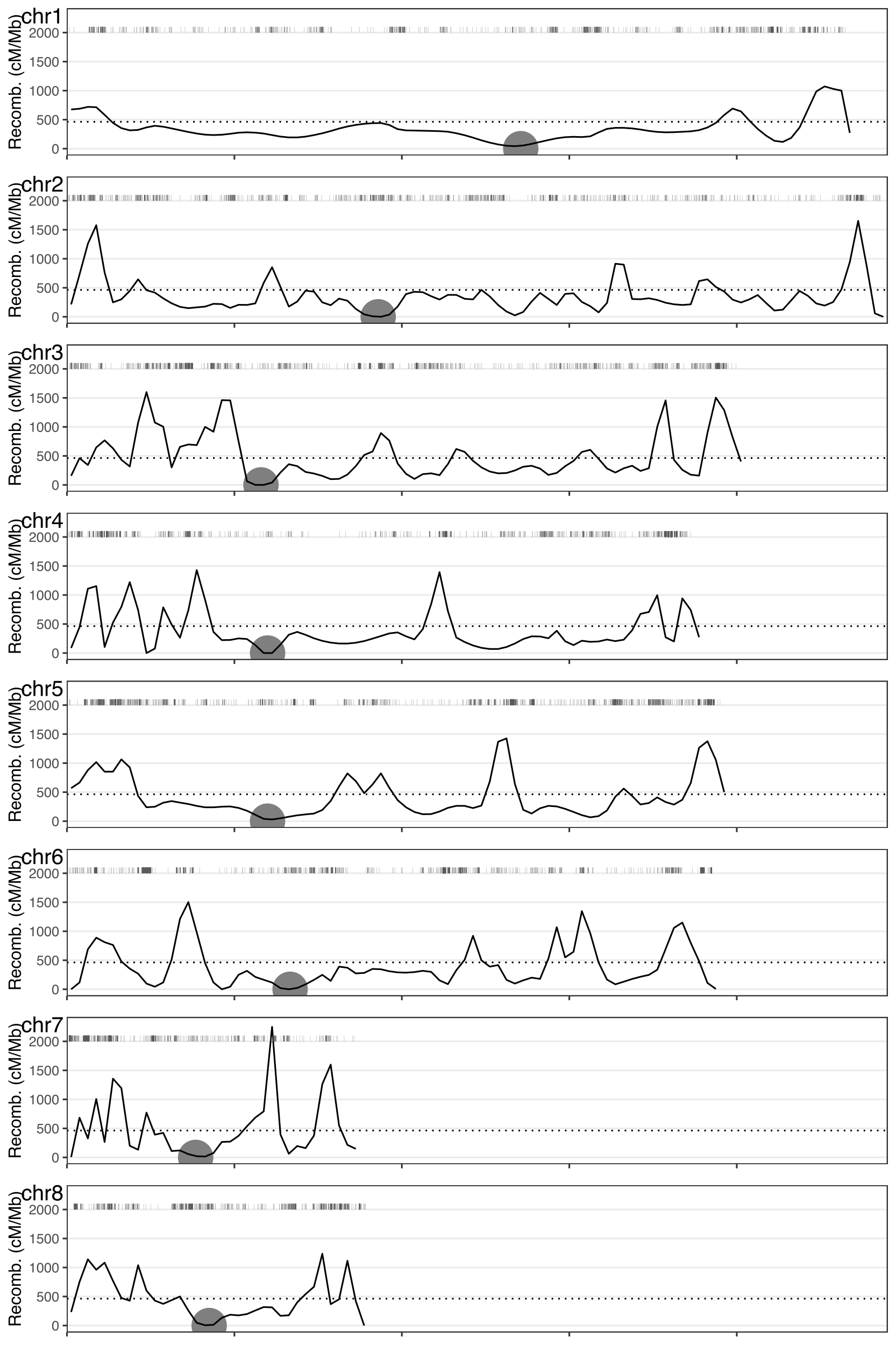


**Fig. S5. Recombination landscapes of all eight chromosomes of *A. fumigatus*.** Plots are similar to Figure 2B of the main text but shown for all eight chromosomes. Dotted line indicates the genome-wide average recombination rate, solid line indicates recombination rate across 50kb windows. Small vertical hash lines at top of plots indicate marker positions, and large circle indicates estimated centromere position.
