# Supplementary material for: The human fungal pathogen Aspergillus fumigatus can produce the highest known number of meiotic crossovers
Source: PLoS Biol. 2023 Sep 14;21(9):e3002278. doi: 10.1371/journal.pbio.3002278 (PMC10501685; doi:10.1371/journal.pbio.3002278)
Supplement: S6 Fig — (A) Whole contents of single cleistothecia plated on Malt Extract Agar plates. (B) Whole contents of a single cleistothecium selected for cyp51A recombinants by plating instead on MEA + 10 μg/mL itraconazole. Bottom right plate is the selection plate shown in Fig 4. (DOCX) [file pbio.3002278.s006.docx]

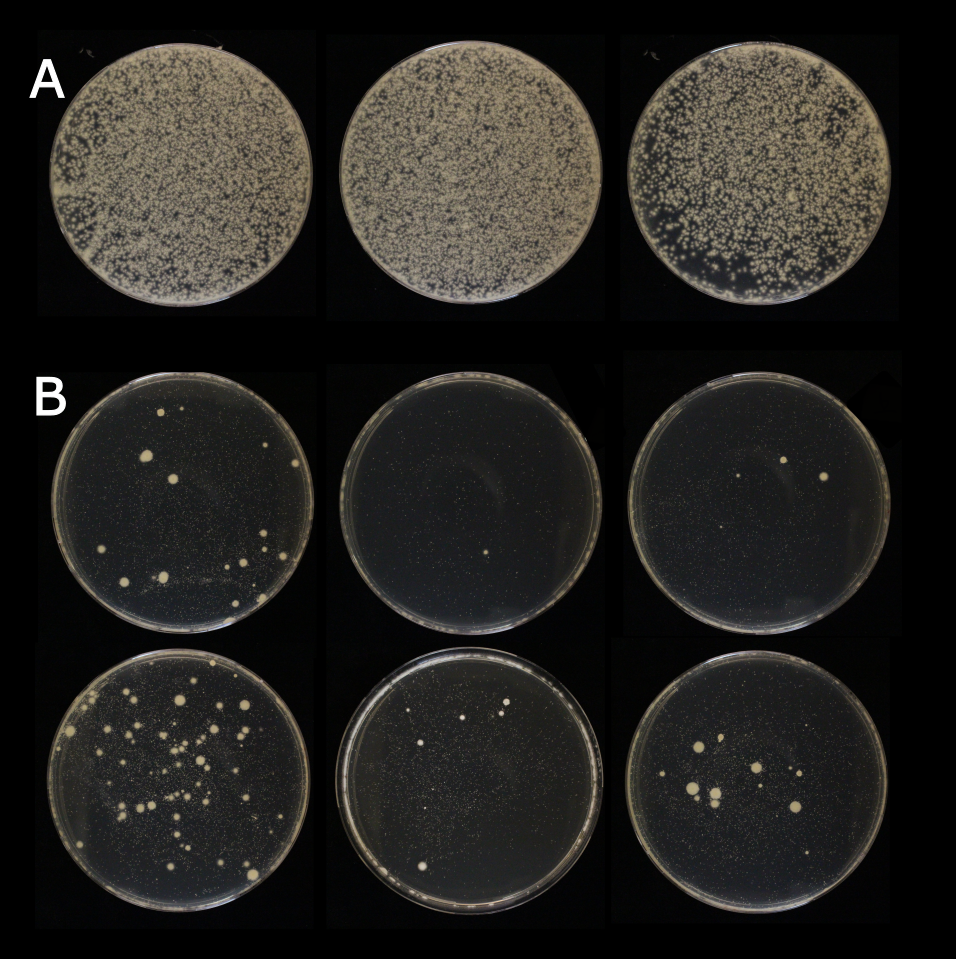


**Figure S6:** **Intragenic *cyp51A* recombination in *A. fumigatus* ascospore progeny.** **(A)** Whole contents of single cleistothecia plated on Malt Extract Agar plates. **(B)** Whole contents of a single cleistothecium selected for *cyp51A* recombinants by plating instead on MEA + 10 µg/mL itraconazole. Bottom right plate is the selection plate shown in Fig. 4.
