# Supplementary material for: The human fungal pathogen Aspergillus fumigatus can produce the highest known number of meiotic crossovers
Source: PLoS Biol. 2023 Sep 14;21(9):e3002278. doi: 10.1371/journal.pbio.3002278 (PMC10501685; doi:10.1371/journal.pbio.3002278)
Supplement: S1 Table — Data underling this figure can be found at https://doi.org/10.5281/zenodo.8167717. (DOCX) [file pbio.3002278.s007.docx]

**Table S1: Parental genome assembly statistics**

| Sequence Data | | |  | Final Assembly | | |
| --- | --- | --- | --- | --- | --- | --- |
|  | AfIR974 | AfIR964 |  | Chromosome | AfIR974 (P0) | AfIR964 (P1) |
| Nanopore Data N50 | 25.2 kb | 11.4 kb |  | Chr1 | 4,663,655 | 4,668,212 |
| Nanopore Depth | 107X | 88X |  | Chr2 | 4,867,745 | 4,879,980 |
| minimap2/miniasm contigs | 10 | 22 |  | Chr3 | 4,028,444 | 4,004,818 |
| canu/racon contigs | 13 | 16 |  | Chr4 | 3,769,667 | 3,715,983 |
| BUSCO analysis | | |  | Chr5 | 3,924,433 | 3,954,489 |
| Complete | 4000 | 4017 |  | Chr6 | 3,858,511 | 3,795,227 |
| Complete single-copy | 3996 | 4013 |  | Chr7 | 1,723,905 | 1,756,011 |
| Complete duplicated | 4 | 4 |  | Chr8 | 1,792,146 | 1,743,900 |
| Fragmented | 21 | 10 |  | Mitochondrion | 30,692 | 31,032 |
| Missing | 25 | 19 |  | **Total** | **28,659,198** | **28,549,652** |
| Total | 4046 | 4046 |  |  |  |  |
