# Supplementary material for: The human fungal pathogen Aspergillus fumigatus can produce the highest known number of meiotic crossovers
Source: PLoS Biol. 2023 Sep 14;21(9):e3002278. doi: 10.1371/journal.pbio.3002278 (PMC10501685; doi:10.1371/journal.pbio.3002278)
Supplement: S2 Table — Data underling this figure can be found at https://doi.org/10.5281/zenodo.8167717. (DOCX) [file pbio.3002278.s008.docx]

**Table S2: Types of variants and their effects.**

|  | Variant type | | | |
| --- | --- | --- | --- | --- |
| Effect of variant | Multi-nucleotide variant | Insertion/deletion | Single nucleotide variant | sum |
| Non-synonymous | 146 | 67 | 2,212 | 2,425 |
| Intergenic | 829 | 597 | 8,209 | 9,635 |
| Intron | 2 | 20 | 143 | 165 |
| Synonymous | 18 | 0 | 1,870 | 1,888 |
| summed type | 995 | 684 | 12,434 | 14,113 |
