# Supplementary material for: The human fungal pathogen Aspergillus fumigatus can produce the highest known number of meiotic crossovers
Source: PLoS Biol. 2023 Sep 14;21(9):e3002278. doi: 10.1371/journal.pbio.3002278 (PMC10501685; doi:10.1371/journal.pbio.3002278)
Supplement: S3 Table — From left to right, chromosome, chromosome length, number of variants detected, recombination events per offspring, raw recombination fraction, rarefied recombination fraction, and gene conversion (GC) corrected map length is shown. Data underling this figure can be found at https://doi.org/10.5281/zenodo.8167717. (DOCX) [file pbio.3002278.s009.docx]

**Table S3: Genetic map length statistics**. From left to right, chromosome, chromosome length, number of variants detected, recombination events per offspring, raw recombination fraction, rarefied recombination fraction and gene conversion (GC) corrected map length is shown.

| Chr | Length (bp) | Number of variants | Mean recombination events / offspring | cM recombination fraction | cM rarefied recombination fraction (35 cM) | cM GC corrected |
| --- | --- | --- | --- | --- | --- | --- |
| 1 | 4,663,655 | 1,270 | 16.59 | 1,916 | 1,663 | 1,654 |
| 2 | 4,867,745 | 1,685 | 20.56 | 2,222 | 1,639 | 1,704 |
| 3 | 4,028,444 | 2,061 | 22.51 | 2,509 | 1,952 | 1,887 |
| 4 | 3,769,667 | 2,107 | 16.63 | 1,871 | 1,448 | 1,429 |
| 5 | 3,924,433 | 2,265 | 18.44 | 2,025 | 1,524 | 1,465 |
| 6 | 3,858,511 | 2,015 | 17.64 | 2,058 | 1,685 | 1,615 |
| 7 | 1,723,905 | 1,254 | 9.95 | 1,127 | 929 | 872 |
| 8 | 1,792,146 | 1,456 | 9.87 | 1,115 | 857 | 899 |
| sum | 28,628,506 | 14,113 | 132.18 | 14,848 | 11,698 | 11,525 |
