# Supplementary material for: The human fungal pathogen Aspergillus fumigatus can produce the highest known number of meiotic crossovers
Source: PLoS Biol. 2023 Sep 14;21(9):e3002278. doi: 10.1371/journal.pbio.3002278 (PMC10501685; doi:10.1371/journal.pbio.3002278)
Supplement: S4 Table — All parental isolates are azole sensitive Dutch or Irish environmental isolates unless specified otherwise. (DOCX) [file pbio.3002278.s010.docx]

**Table S4: Details of crosses used for recombination analyses.** All parental isolates are azole sensitive Dutch or Irish environmental isolates unless specified otherwise.

| Cross | Parents | Number of offspring sequence | Number of filtered segregating markers | Genetic map length* (cM) |
| --- | --- | --- | --- | --- |
| Original | AfIR974 X AfIR964 | 195 | 14,113 | 12,469 |
| UK1 | AfIR974 X 47-55 | 12 | 48,653 | 5,778 |
| UK2 | C78** X 47-55 | 15 | 36,898 | 4,436 |
| NL1 | 88C19 X 46A23 | 7 | 40,579 | 18,013 |

*To reduce the effect of gene conversions, map length was estimated after rarefaction of markers to 20 cM spacing, as in Figure 1A of the main text.

**Isolate C78 is an azole resistant isolate of *cyp51A* TR_34_/L98H genotype.
