# Supplementary material for: The human fungal pathogen Aspergillus fumigatus can produce the highest known number of meiotic crossovers
Source: PLoS Biol. 2023 Sep 14;21(9):e3002278. doi: 10.1371/journal.pbio.3002278 (PMC10501685; doi:10.1371/journal.pbio.3002278)
Supplement: Attachment — Submitted filename: Response to Reviewers.docx [file pbio.3002278.s011.docx]

Please also address the following policy and formatting requests:

**1. DATA POLICY:**

You may be aware of the PLOS Data Policy, which requires that all data be made available without restriction: <http://journals>.plos.org/plosbiology/s/data-availability. For more information, please also see this editorial: <http://dx.doi.org/10.1371/journal.pbio.1001797>

Note that we do not require all raw data. Rather, we ask that all individual quantitative observations that underlie the data summarized in the figures and results of your paper be made available in one of the following forms:

A) Supplementary files (e.g., excel). Please ensure that all data files are uploaded as 'Supporting Information' and are invariably referred to (in the manuscript, figure legends, and the Description field when uploading your files) using the following format verbatim: S1 Data, S2 Data, etc. Multiple panels of a single or even several figures can be included as multiple sheets in one excel file that is saved using exactly the following convention: S1_Data.xlsx (using an underscore).

B) Deposition in a publicly available repository. Please also provide the accession code or a reviewer link so that we may view your data before publication.

Regardless of the method selected, please ensure that you provide the individual numerical values that underlie the summary data displayed in the following figure panels as they are essential for readers to assess your analysis and to reproduce it: Figures 1ABCDEFG, 2ABCDEF, 3BC, 4B, Supplementary Figures S1ABC, S2AB, S3ABCDEFGHI, S4AB, S5.

NOTE: the numerical data provided should include all replicates AND the way in which the plotted mean and errors were derived (it should not present only the mean/average values).

**Please also ensure that figure legends in your manuscript include information on where the underlying data can be found, and ensure your supplemental data file/s has a legend.**

Please ensure that your Data Statement in the submission system accurately describes where your data can be found.

*We have chosen to use option B above (https://doi.org/10.5281/zenodo.8162915), and uploaded our data as an accession to Zenodo, with a link in our Data Availablilty statement. For our figures, the values are derived within an R script after processing the genotypic and phenotypic data. Thus, the data underlying the figure is available, but we have not produced intermediate values for each plot/panel.*

**2.** Please note that sole deposition of data or code to GitHub would not be compliant with our policies, as this could be changed after publication (<https://journals>.plos.org/plosbiology/s/data-availability). However, once the data/code is final, you can archive your publicly available GitHub data to Zenodo. Once you do this, it will also generate a DOI number that you can provide us with. See the process for doing this here: 
<https://docs.github.com/en/repositories/archiving-a-github-repository/referencing-and-citing-content>

*We have created a Zenodo repository from our Github code (https://doi.org/10.5281/zenodo.8167717).*
**3.** We suggest a change in the title: "The human fungal pathogen Aspergillus fumigatus can produce the highest known number of meiotic crossovers".

*This has been updated*

We expect to receive your revised manuscript within 1 month. Please email us (plosbiology@plos.org) if you have any questions or concerns, or would like to request an extension.

At this stage, your manuscript remains formally under active consideration at our journal; please notify us by email if you do not intend to submit a revision so that we withdraw the manuscript.

**IMPORTANT - SUBMITTING YOUR REVISION**

Your revisions should address the specific points made by each reviewer. Please submit the following files along with your revised manuscript:

1. A 'Response to Reviewers' file - this should detail your responses to the editorial requests, present a point-by-point response to all of the reviewers' comments, and indicate the changes made to the manuscript.
*NOTE: In your point-by-point response to the reviewers, please provide the full context of each review. Do not selectively quote paragraphs or sentences to reply to. The entire set of reviewer comments should be present in full and each specific point should be responded to individually.
You should also cite any additional relevant literature that has been published since the original submission and mention any additional citations in your response.

2. In addition to a clean copy of the manuscript, please also upload a 'track-changes' version of your manuscript that specifies the edits made. This should be uploaded as a "Revised Article with Changes Highlighted " file type.

*Resubmission Checklist*
When you are ready to resubmit your revised manuscript, please refer to this resubmission checklist: https://plos.io/Biology_Checklist

To submit a revised version of your manuscript, please go to <https://www.editorialmanager.com/pbiology/>and log in as an Author. Click the link labelled 'Submissions Needing Revision' where you will find your submission record.

Please make sure to read the following important policies and guidelines while preparing your revision:

*Published Peer Review*
Please note while forming your response, if your article is accepted, you may have the opportunity to make the peer review history publicly available. The record will include editor decision letters (with reviews) and your responses to reviewer comments. If eligible, we will contact you to opt in or out. Please see here for more details:
<https://blogs>.plos.org/plos/2019/05/plos-journals-now-open-for-published-peer-review/

*PLOS Data Policy*
Please note that as a condition of publication PLOS' data policy (<http://journals>.plos.org/plosbiology/s/data-availability) requires that you make available all data used to draw the conclusions arrived at in your manuscript. If you have not already done so, you must include any data used in your manuscript either in appropriate repositories, within the body of the manuscript, or as supporting information (N.B. this includes any numerical values that were used to generate graphs, histograms etc.). For an example see here: <http://www>.plosbiology.org/article/info%3Adoi%2F10.1371%2Fjournal.pbio.1001908#s5

*Blot and Gel Data Policy*
We require the original, uncropped and minimally adjusted images supporting all blot and gel results reported in an article's figures or Supporting Information files. We will require these files before a manuscript can be accepted so please prepare them now, if you have not already uploaded them. Please carefully read our guidelines for how to prepare and upload this data: <https://journals>.plos.org/plosbiology/s/figures#loc-blot-and-gel-reporting-requirements

*Protocols deposition*
To enhance the reproducibility of your results, we recommend that if applicable you deposit your laboratory protocols in protocols.io, where a protocol can be assigned its own identifier (DOI) such that it can be cited independently in the future. Additionally, PLOS ONE offers an option for publishing peer-reviewed Lab Protocol articles, which describe protocols hosted on protocols.io. Read more information on sharing protocols at https://plos.org/protocols?utm_medium=editorial-email&utm_source=authorletters&utm_campaign=protocols


Thank you again for your submission to our journal. We hope that our editorial process has been constructive thus far, and we welcome your feedback at any time. Please don't hesitate to contact us if you have any questions or comments.

Sincerely,
Paula

---
Paula Jauregui, PhD,
Senior Editor
PLOS Biology
pjaureguionieva@plos.org

----------------------------------------------------------------

REVIEWS:


Reviewer #1: Genome dynamics.

Reviewer #2: Chromosomes, mitosis and meiosis.

Reviewer #3: Meiosis recombination.


Reviewer #1: Auxier et al. characterized meiotic recombination events genome wide in the progeny of four Aspergilus fumigatus intraspecific hybrids. In two of them, they found the highest meiotic crossover frequency ever recorded, in agreement with the very short LD50 previously described in this species. This property is not shared with the two other hybrids studied, indicating a high variability in crossover frequency within this species. The authors further took advantage of this elevated meiotic crossover frequency for precise QTL mapping, and to generate intragenic recombined offspring at the cyp51A locus.

Given the novelty of their main finding, the elevated recombination frequency of A. fumigatus, the authors took great care of challenging it with stringent criteria to make sure it was not artifactual (ie coming for gene conversion events not associated with any crossover).

Overall, the results are convincing and bring new and interesting light on the variability of the regulation of meiotic recombination, identifying a new "world champion" with higher crossover number per chromosome than the former champions that are Schizosaccharomyces pombe and Saccharomyces cerevisiae.

Comments

1. The introduction is so minimal that it is even shorter than the abstract that contains more introductive information: this cannot stand as it is. The authors should provide a review of the literature about Aspergilus fumigatus meiotic recombination.

*We have added two introductory paragraphs with information about recombination rate, meiotic biology, and a comparison of different Aspergillus* *species. We have expanded the Introduction of the manuscript to include additional details about the sexual cycle both in A. fumigatus as well as compared to other Aspergillus species.*

2. Unlike in budding and fission yeasts where the four gametes per meiosis can be easily isolated for genotyping, here a main limitation to the analysis is that it relies on random isolation of haploid offspring. Therefore, crossover identification relies on only half of the recombined material, the other complementary /reciprocal half being unavailable for genotyping. I would like the authors to consider this as a possible caveat to this strategy since crossovers are not directly seen but inferred.

*We agree with the reviewer that when four (or eight) gametes can be isolated separately such an analysis is of great value. In contrast to budding and fission yeast, where the tetrads are the end product of meiosis and can relatively easily be harvested, in A. fumigatus the eight asci stage during meiosis is a temporary state in the meiosis cycle with an unknown timing. Also, technically it is not yet feasible to harvest these fragile asci. Inferring crossovers is therefore at this point the only feasible analysis that can be performed.*

I acknowledge that I am not a specialist of A. fumigatus meiosis. From what I understand, gamete formation takes 4-6 weeks. Is it known for sure that during this time, only one meiosis takes place during gamete formation? What if several meioses take place during this time in combination with spore mating? Can this possibility be
 out? Alternatively, in budding yeast the mechanism of return to growth (RTG) has been described and consists of an abortive meiosis after Spo11-DSBs are formed. In case several rounds of RTG take place prior full meiosis completion, the number of crossover sites determined from the haploid offspring will look artificially high compared to the actual number of crossover sites per meiosis.

*The sexual ascospores enter quite a dormant state, and no return to growth has ever been observed to these authors’ knowledge. In the absence of the 70C heat shock (Zhang et al. 2022:* [*https://doi.org/10.3389/fcimb.2021.785157*](https://doi.org/10.3389/fcimb.2021.785157)*), the spores germinate quite poorly, so such a mechanism seems unlikely.*

3. Given the SNP density, simple gene conversions not associated to any crossover (ie non-crossovers) should be readily identifiable. Although somewhat mentioned along the text, it looks to me that a specific paragraph should be dedicated to the frequency of non-crossovers, their putative properties (length etc).

*We have added a short description of what we think can be learned about gene conversions in* A. fumigatus*. We have not added many details, as without tetrad analysis, we are hesistant to strictly delineate gene conversion events from close double crossovers.*

Furtheremore, the definition of non-crossover / gene conversion should be revised. Gene conversion result from the repair of mismatches that are formed in any recombination event, whether it gives rise to a crossover or a non-crossover.

*We have updated our definition near the bottom of page 3 accordingly.*

4. Unsean double CO (CO = crossover). Although heterogenous, the marker density is such that it leaves very little room for unsean double CO.

*Although the marker density is high, there are many large regions of identical sequence. For example, surrounding cyp51A, there is an approximately 20kb stretch of identical sequence. Such regions are large enough that double crossovers are possible, when assuming a crossover occurring approximately once per 100kb of sequence.*

5. The reference for non-crossover / gene conversion in A. fumigatus is Chen et al 2007 which is not specific to A. fumigatus. What is known about gene conversions and gene conversion tract length in A. fumigatus?

*We have clarified that this reference is to gene conversions in general, not specifically regarding to A. fumigatus. As our manuscript is the first report of a genetic analysis for A. fumigatus, such details are only available now in our data. Even for additional species, like A. nidulans* *and A. flavus, while genetic maps are available, they are based on marker analyses, not whole genomic sequences. Therefore, there is no information on conversion tract lengths from any Aspergillus species as far that we are aware of.*

6. Some clarification is needed in the comparison of the recombination properties of A. fumigatus with respect to published data and reference species such as S. cerevisiae.
Considering 132 CO per haploid offspring, it is expected to see about 264 CO per meiosis in A. fumigatus, ie 264 CO / 30 Mb ie 8.8 CO per Mb.
In S. cerevisiae, Mancera et al. reported ca. 90 CO per meiosis, ie 90 CO / 12 Mb ie 7.5 CO per Mb.
In conclusion, the number of CO per meiosis is clearly higher in A. fumigatus than is S. cerevisiae, but the number of CO per Mb is comparable between the two species. Given the strict correlation between chromosome size and CO number in S. cerevisiae, it looks like the same applies to A. fumigatus, but with a higher total CO number per meiosis resulting from a longer genome.

*It is unclear to us what modifications/clarification the reviewer is specifically asking for in this comment. Comparing against S. cerevisiae has the confounding factor of twice the number of chromosomes than A. fumigatus, and so it would be just as reasonable to provide the numbers as 256 “excess” CO for fumigatus, and 74 “excess” for cerevisiae, since one crossover is necessary per chromosome. While this is a small change, it changes the numbers to 8.7 CO/Mb for fumigatus, and 6.1 CO/Mb for cerevisiae. Thus, fumigatus has 40% more “excess” crossovers per Mb of sequence, not an insubstantial fraction. Additionally, baker’s yeast has a genome that is famously streamlined for a eukaryote, basically without introns, and with small intergenic spaces and few introns. So, a CO/Mb is not as biologically meaningful. We could just as easily calculate the CO/gene, where fumigatus would produce 264 CO/9915 genes, while cerevisiae would produce 90 CO/6465 genes.*

*In summary, while there are many possible metrics that can be evaluated, we provide the first genetic data for fumigatus. This allows comparisons against whatever species a reader may find interesting.*

7. Figure 2F. The axes annotations do not match with the legend of the figure. Double check.

*We have updated the legend to clarify, but this graph is meant to show the correction between the number of crossovers occurring on Chr #1 versus the total number per individual (excluding #1), plotted per individual. This data recapitulates the previous finding of correlated crossovers occurring per meiosis, shown by Wang et al. 2019 which we cite in the main text.*

8. CO interference:
Although S. pombe is considered as lacking CO interference, work from G. Smith lab revealed some short-range CO interference likely resulting from DSB spacing, controlled by Tel1.
Interestingly, J. Fung lab revealed that the absence of Tel1 also impacts on CO interference in S. cerevisiae, suggesting part of CO interference in S. cerevisiae results from DSB spacing.
Finally, recent work done in the budding yeast *Lachancea waltii* revealed the existence of a weak short-range interference independent of the ZMM CO interference pathway.
In conclusion, I suggest the authors double check their analysis of CO interference to specifically determine if they also detect such a short-range CO interference which would also likely result from the DSB spacing.
*This would indeed be an interesting topic to study. Whether our lack of observed interference truly means COs are independent even at small scales is an interesting question. Unfortunately, in the absence of tetrad data, we do feel such close analyses are feasible with our data. Particularly for the shorter inferred double crossovers events, the chance of a misclassification with a gene conversion tract is particularly high.*

9. Figure 2B and S5.
In most species studied so far, CO are suppressed around centromeres. Here, chromosome 1 is used as a representative chromosome in Fig 1B, but it is the only chromosome not showing a CO depletion around the centromere, while all the seven other chromosomes show a depletion around it.
I therefore suggest using another chromosome for Fig 2B in order to be as representative as possible.
However, I am surprised to see a CO coldspot on chromosome 1 ca 0.7MB right of the centromere. I suggest the authors double check for the precise annotation of the centromere of chromosome 1, and whether it is annotated at the right place.
*Indeed, we had Chr 1 plotted reversed, and we would like to thank the reviewer for identifying this. In our assembly it is reversed from the Af293 orientation, so this led to an error in the R script plotting the final figure. We have corrected this.*

Typo:
Is occurring "in" azole-contaminated bulb waste heaps

*fixed*

Reviewer #2: This work from Auxier et al presents interesting and useful observations that should be of interest to the readers of PLoS Biology.

I have only two comments.

1. Title: ...."the highest known number of crossovers". The highest known number of crossovers per what? Per Mb? Per chromosome? Per genome? From the abstract it seems that the authors are interested in the number per chromosome (per pair of homologs). But the text emphasizes the number of crossovers per total genome size in Mb (Figure 1F) and the number of crossovers per Mb per chromosome (Figure 1G). The authors seem to be mostly interested in the genetic consequences of crossing-over, in which case crossovers per Mb is most directly relevant and the title should emphasize this feature. The absolute number of crossovers per chromosome is of interest if one is interested in the role of crossing-over in meiotic chromosome segregation, but this is not the authors' focus. The number per genome is less interesting without reference to total genome size and/or total chromosome number.

*We have taken the suggestion of the Editor’s office regarding modifications to the title of the manuscript.*

2. The authors understanding of the mechanics of meiosis should be improved.
- "To some extent, the high numbers of crossovers that we observe may be explained as the lack of the SC required to ensure that at least one crossover occurs per chromosome pair, necessary for proper meiotic chromosome reduction (Egel-Mitani et al., 1982; Snow, 1979)."
The two references cited are from 1979 and 1982. A lot has happened since then. In two other fungi (budding yeast and Sordaria macrospora) SC is not required, mechanistically, to ensure that at least one crossover occurs per chromosome pair and, in both cases, is the downstream outcome of the processes that determine crossover numbers and interference.
- "To some extent, the high numbers of crossovers that we observe may be explained as the lack of the SC required to ensure that at least one crossover occurs per chromosome pair, necessary for proper meiotic chromosome reduction (Egel-Mitani et al., 1982; Snow, 1979)." The authors might want to read the Arabidopsis literature which identifies genetic situations in which the numbers of crossovers are dramatically increased, and those crossovers don't exhibit interference. Perhaps Aspergillus fumigatus is an analogous mutant case, particularly since the "two strains" in the cross are not genetically identical, so the high level of crossovers could be a consequence of genetic heterozygosity (irrespective of structural variations). Ref: Serra H, Lambing C, Griffin CH, Topp SD, Nageswaran DC, Underwood CJ, Ziolkowski PA, Séguéla-Arnaud M, Fernandes JB, Mercier R, Henderson IR. Proc Natl Acad Sci U S A. 2018.

*Although we had tried to limit the speculation regarding the relationship between the potential lack of a SC and this increased recombination rate, it seems we were too hesitant. We have added discussion of the papers/mechanisms to the near the bottom of page 11.*

*Regarding the relationship of our experiment to field settings, since A. fumigatus is an obligate outcrossing (heterothallic) species, meiosis does not occur between genetically identical genomes in the field. The parental strains we used are not more genetically diverse than expected for this species (extensive sampling of Dutch isolates indicates between 20,000 to 50,000 variants between any random selected pair, when using less stringent filtering. Data from a forthcoming manuscript: Snelders et al.* in prep*). Further, genetic heterozygosity has been demonstrated to decrease crossing over, as it reduces the ability for homology detection (Chen and Jinks-Robertson, 1999* <https://doi.org/10.1093/genetics/151.4.1299>*). While mutants in different species indeed produce an increased number of non-interfering crossovers, we are discussing the evolutionary maintenance of such a crossover number. If this species perhaps did lose some of the functionality compared to other Aspergilli, we feel it is still interesting to consider what forces may maintain such high crossover numbers.*

Reviewer #3: This is truly an excellent paper. The authors have been extremely careful in their analysis. I was especially impressed by the manner in which they coped with the complexity of gene conversion. I was initially a bit worried about an absence of a perspective of 'meiotic biology - specifically, the lack of interference and the absence of the SC'. But the authors addressed these issues beautifully, along with the appropriate comparison to S pombe, on page 6. My one suggestion would be to encourage the authors to make reference of this discussion of meiotic biology in their Abstract. It is important.

The paper is a lovely contribution to several fields and has tangible application to public health.

This a very good paper and I strongly encourage publication. I feel badly that I could not find any significant flaw. I tried. But it is a really nice paper!

*We thank the reviewer for the kind words and have added a few words to the abstract to emphasize the meiotic biology accordingly.*
